# Modeling the compaction of bacterial chromosomes by biomolecular crowding and the cross-linking protein H-NS

**DOI:** 10.1038/s41598-023-50355-2

**Published:** 2024-01-02

**Authors:** Youngkyun Jung, Amir Sadeghi, Bae-Yeun Ha

**Affiliations:** 1 Supercomputing Center, Korea Institute of Science and Technology Information, Daejeon, 34141 South Korea; 2 Department of Physics and Astronomy, University of Waterloo, Waterloo, ON N2L 3G1 Canada

**Keywords:** Biological physics, Chemical physics

## Abstract

Cells orchestrate the action of various molecules toward organizing their chromosomes. Using a coarse-grained computational model, we study the compaction of bacterial chromosomes by the cross-linking protein H-NS and cellular crowders. In this work, H-NS, modeled as a mobile “binder,” can bind to a chromosome-like polymer with a characteristic binding energy. The simulation results reported here clarify the relative role of biomolecular crowding and H-NS in condensing a bacterial chromosome in a quantitative manner. In particular, they shed light on the nature and degree of crowder and H-NS synergetics: while the presence of crowders enhances H-NS binding to a chromosome-like polymer, the presence of H-NS makes crowding effects more efficient, suggesting two-way synergetics in chain compaction. Also, the results show how crowding effects promote clustering of bound H-NS. For a sufficiently large concentration of H-NS, the cluster size increases with the volume fraction of crowders.

## Introduction

**Figure 1:**
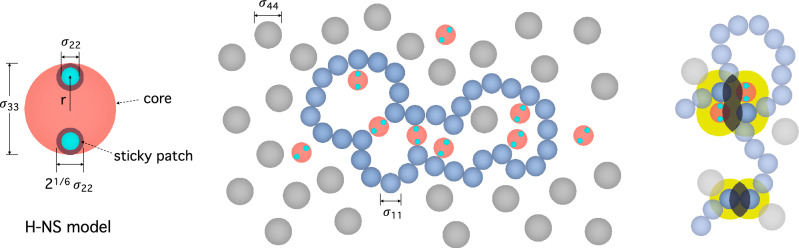
H-NS model and chromosome-like polymer in a crowded medium containing H-NS. As shown on the left, H-NS is a triplex, consisting of a core (red-orange) and two patches (cyan) on the opposite sides of the core. The patch interacts with the polymer only; the shaded region around a patch sphere represents the interaction range. The spatial organization of the polymer is influenced by both crowding effects and the binding of H-NS, as illustrated in the middle. The role of H-NS in condensing DNA is two-fold: cross-linking or bridging and enhancement of crowding effects. As shown at the bottom right, each monomer can be viewed as being surrounded with a depletion layer (in yellow) inside which the center of crowders are excluded. Overlapping of depletion layers increases the space available to crowders. Association of monomers or two molecules is favored by the entropy of crowders. This is the origin of depletion forces. Also, the binding of H-NS leads to an enlarged depletion layer in yellow, as shown at the top right. The degree of overlapping between two such layers (shades) essentially determines the strength of depletion forces: the larger the shade is, the stronger the depletion force is. As a result, the binding of H-NS can amplify the effect of crowding on chain compaction. Here $\sigma _{11}\equiv \sigma$ (monomer size), $\sigma _{22}$ (H-NS patch size), $\sigma _{33}=\sigma$ (H-NS core size), and $\sigma _{44}\equiv \sigma _c$ (crowder size).

Chromosomes in cells are tightly packed but maintain a high level of organization^1–3^. What holds them in organized structures, as required for their biological functions (e.g. transcription or replication, ...)? Indeed, chromosome organization is a challenging task every cell faces and relies on the action of various molecules and the physical effects they bring about^1–3^. In the case of bacterial chromosomes, a number of studies clarify the roles of nucleoid associated proteins (NAPs) such as HU and H-NS^4–7^. They bend, cross-link, loop, or supercoil the chromosomal DNA. Also, the significance of biomolecular crowding has been highlighted^2,8–15^. In a crowded cellular space^8,16,17^, chain molecules such as chromosomes can be entropically collapsed and phase-separated from the surrounding crowders^2,8–15^. As shown at the bottom right in Fig. 1, chain segments experience entropic (depletion) attractions induced by crowding effects^18^. Each monomer can be viewed as being surrounded with a ‘depletion layer’ (shown in yellow) inside which the center of crowders are excluded^18^. Overlapping of depletion layers increases the space available to crowders. As a result, association of monomers is favored by the entropy of crowders. This is the origin of depletion forces.

While relying heavily on this non-specific (entropic) mechanism, bacterial chromosome organization should be controlled in such a way that it works in concert with other processes, such as transcription, chromosome segregation, and cell growth^2,3,19^. Furthermore, a number of recent studies point to the synergetic effects brought about by crowders and the protein H-NS^20,21^. H-NS alone has a relatively-minor effect on chromosome compaction, similarly to what other chromosome-associating proteins do^14^, but its effect can be magnified in the presence of crowders.

H-NS is one of about 12 NAPs best known for *E. coli*. It is abundantly present at $\sim 20000$ copies per genome equivalent or at the concentration of $\sim 20\upmu \text{M}$^4–6^. It forms a dimer and binds preferentially curved (otherwise non-specifically) DNA, which is characteristic of promoters; it is a transcriptional repressor^4,6^. Also, it condenses bacterial DNA by cross-linking two sites on DNA, which are possibly distant along the DNA^4,6,7,22,23^. Together with other NAPs, H-NS is responsible for small-scale ($\le 1 \, \text{kb}$) DNA compaction^4,7^.

Along this line of the discussion on H-NS and crowder synergetics above, it is worth mentioning that depletion interactions are size-dependent^2,9,12,15^. First, the effect of crowders on chain compaction is marginal when the crowders are bigger in size than monomers^12,15^. The typical size of cellular crowders ($\approx 5 \, \text{nm}$) is larger than the thickness of DNA ($\approx 2 \, \text{nm}$). Nevertheless, crowding is known to be the main player in condensing bacterial chromosomes^11,14^. Indeed, recent studies show that the binding of molecules to an otherwise homogeneous polymer can make more effective chain compaction by crowding effects, as is particularly the case for bacterial chromosome compaction^9^. This aligns well with the aforementioned H-NS and crowder synergetics^20,21^.

Despite much effort, a clear physical picture of the interplay between H-NS and crowders is still elusive. To explain the aforementioned synergetics, a mean-field type approach was employed, in which the binding of H-NS to DNA was viewed as thickening the DNA^20^. In a computational approach^24^, H-NS was adequately modeled as a mobile binder but crowders were not included. In the absence of crowders, the cross-linking activity of H-NS can be mimicked by sparse bridging attractions between designated monomers dispersed along the chain backbone^25^. A better understanding of the interplay between cross-linking and crowding effects would necessitate a more systematic consideration of the interplay under controlled conditions without suppressing important details such as crowders, the association of H-NS with DNA, and the interactions between H-NS and crowders.

Here, using coarse-grained molecular dynamics simulations based on polymer physics, we study the compaction of bacterial chromosomes by H-NS and crowders. Indeed, much of the recent progress in understanding chromosome organization is owed to polymer models of chromosomes^2,9–11,13,24–28^. In this work, we build on recent modeling efforts^2,9,10,13,24^. To this end, we consider a ring polymer interacting with mobile binders in a crowded space. Each binder, modeled as a dimer with two binding sites, can bridge two segments of the polymer, which are possibly distant along the backbone; as detailed below, for this, we follow the modeling strategy proposed in Ref.^24^. Its effect mimics the action of H-NS on bacterial chromosomes.

The simulation results reported here clarify the relative significance of biomolecular crowding and H-NS in chromosome compaction in a quantitative manner. In particular, they offer a clear picture of crowder and H-NS synergetics beyond recent efforts^20,21^. The general picture emerging from our work can be summarized as follows. While the presence of crowders enhances H-NS binding to a chromosome-like polymer by increasing the cross-linking tendency of H-NS, the presence of H-NS makes crowding effects more efficient. This interdependence suggests two-way synergetics between H-NS and crowders in chromosome compaction. Furthermore, our results suggest that crowding effects can promote clustering of bound H-NS and clarify the conditions under which H-NS forms clusters. For a sufficiently large (biologically relevant) concentration of H-NS ($\gtrsim 20 \, \upmu \text{M}$), H-NS clusters with the cluster size increasing with the volume fraction of crowders.

Our finding of H-NS clustering is reminiscent of oligomerization of H-NS^4,6^. However, it was shown that H-NS dimers can cluster even in the absence of crowders, driven by a bridging-induced attraction between the dimers^24^: when H-NS dimers bridge two parallel DNA strands and form a cluster, the bending energy of DNA and the entropic penalty for DNA looping can be minimized. Nevertheless, our finding based on crowding effects points to the possibility that crowding effects can enhance the oligomerization of H-NS proteins.

## Computational modeling

### Molecular dynamics simulations

Our simulations are aimed at modeling bacterial DNA organization by crowders and the cross-linking protein H-NS. As illustrated in Fig. 1, the DNA is modeled as a string of spherical monomers and crowders as simple spheres as in other studies^9,10,23–25^. As in Ref.^24^, H-NS is modeled as a sphere with circular “sticky” patches on the south and north poles of the sphere, as shown on the left in Fig. 1. It is only the sticky patch that can be nonspecifically attached to the DNA (see below for details).

In our simulations, all spheres (DNA, H-NS, and crowders) interact with each other through a truncated-shifted Lennard-Jones (LJ) potential^29^, given by

1 $$\begin{aligned} U(r)=\left\{ \begin{array}{ll} U_{\text {LJ}}(r) - U_{\text {LJ}}(r_{\text {c}}) &{} \text{ for }\ r<r_{\text {c}}\\ 0 &{} \text{ otherwise } \end{array}\right. , \end{aligned}$$

where $U_{\text {LJ}}(r)$ is the conventional LJ potential:

2 $$\begin{aligned} U_{\text {LJ}}(r)=4\epsilon _{ij}\left[ \left( \frac{\sigma _{ij}}{r}\right) ^{12}-\left( \frac{\sigma _{ij}}{r}\right) ^{6}\right] . \end{aligned}$$

Here, *r* is the center-to-center distance between two spheres and $r_c$ is a cutoff distance. The parameter $\epsilon _{ij}$ determines the strength of the short-range interactions between two spheres labeled as *i* and *j*; the energy unit is set to $\epsilon =1.0 \,k_{\text {B}}T$, where $k_{\text {B}}$ is the Boltzmann constant and *T* is the absolute temperature. The parameter $\sigma _{ij}$ represents the range of the LJ potential. The subscripts $i, j = 1, 2, 3, 4$ refer to DNA monomers, H-NS patches, H-NS core, and crowders, respectively. For instance, $\sigma _{11} \equiv \sigma$ is the monomer size chosen to be length units; $\sigma _{22}$ is the H-NS patch size; $\sigma _{33}$ is the H-NS core size; and $\sigma _{44}\equiv \sigma _c$ is the crowder size; $\sigma _{12}$ specifies the range of the interaction between a DNA monomer and a H-NS patch in Eq. (2). Other interaction parameters can be interpreted similarly.

DNA monomers are strung together into a chain via the finite extensible nonlinear elastic (FENE) potential between two consecutive monomers,

3 $$\begin{aligned} U_{\text {FENE}}(r)=-\frac{1}{2}k_{0} \left( r_{0}\right) ^{2} \ln \ \left[ 1-\left( \frac{r}{r_{0}}\right) ^{2}\right] .\end{aligned}$$

The spring constant is set to $k_0 = 30 \epsilon / \sigma ^2$ and the range of the potential to $r_0=1.5 \sigma$^30,31^. These choices are to prevent the crossing of monomers.

H-NS is modeled as a complex (triplex) consisting of a core sphere of diameter $\sigma$ and two small patchy spheres of diameter $0.178\sigma$, as noted above. In essence, we follow the modeling strategy in Ref. ^24^. The two patches are embedded into the core sphere and placed near its north and south poles as shown on the left in Fig. 1; their center is $0.4\sigma$ away from the center of the core sphere. This configuration is to avoid multiple contacts between a sticky patch and monomers^24^. To this end, we use the potential energy given by

4 $$\begin{aligned} U_\text {H-NS}(r)=K(r-r_0)^2+k_b(\theta -\pi )^2 \, \end{aligned}$$

where *r* is the center-to-center distance between the core sphere and one of the patches, as shown in Fig. 1 (see the H-NS model on the left). Here $K=120 \epsilon / \sigma ^2$, $r_0=0.4\sigma$ and $k_b=50 \epsilon$; $\theta$ is the angle between two vectors drawn from the center of the core to the two patch spheres. When the patch spheres are on the opposite sides, $\theta =\pi$ as in the H-NS configuration in Fig. 1.

In order to explore the phase space, the equation of motion for monomers is integrated using the velocity-Verlet algorithm with a time step $0.005\tau$. The system is kept at a constant temperature, $T = 1.0 \epsilon /k_{\text {B}}$, via a Langevin thermostat with a damping time, $\tau = \sigma \sqrt{m/\epsilon }$, where *m* is the DNA monomer mass. The choices of *m* is not important in our work because they do not affect equilibrium quantities. For our simulations, we used the simulation package LAMMPS^32^.

We first performed $10^8$ integration steps ($=5\times 10^5 \, \tau$) in order for the system to equilibrate. After equilibration, we ran additionally $10^9$ integration steps ($=5\times 10^6 \, \tau$) and collected data every $5\times 10^3$ time steps ($=25 \, \tau$).

### Choosing the simulation parameters

**Figure 2:**
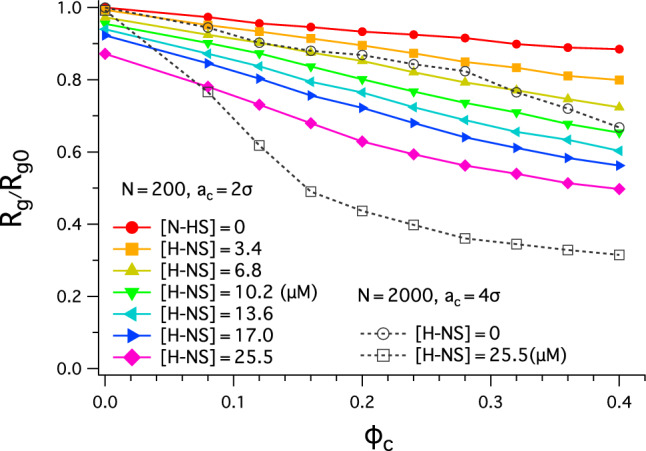
Polymer compaction by molecular crowding for various choices of $[\text {H-NS}]$, the concentration of H-NS dimers. The graph shows the reduced chan size $R_g/R_{g0}$ as a function of $\phi _c$, where $R_g$ is the radius of gyration and $R_{g0}$ is $R_g$ obtained with $\phi _c=0$ and $[\text {H-NS}]=0$. In all cases, the chain size decreases as $\phi$ increases, more rapidly for larger $[\text {H-NS}]$; for given $\phi _c$, it is smaller for larger $[\text {H-NS}]$. When $[\text {H-NS}]=25.5 \, \mu \text {M}$, for example, it is compacted by about $13\%$ and $46\%$ for $\phi _c=0$ and 0.32, respectively. Also shown are curves obtained for a longer chain: $N=2000$. The longer chain is more effectively condensed. When $[\text {H-NS}]=25.5 \, \upmu \text{M}$ and $\phi _c=0.32$, for instance, $R_g/R_{g0} \approx 0.34$.

In our simulations, we chose the parameters as follows. First, we primarily use $N=200$ and $\sigma _{44}=\sigma _c=2 \sigma$ (except in Fig. 2). As a result, the interaction range between a monomer and a crowder becomes $\sigma _{14}=1.5 \sigma$. We also consider a longer chain $N=2000$ with $\sigma _{44}=\sigma _c = 4\sigma$ and $\sigma _{14}= 2.5\sigma$. Recall that the subscripts $i, j = 1, 2, 3, 4$ refer to DNA, H-NS patches, H-NS core, and crowders, respectively. In all cases, $\sigma _{33}=\sigma$ and thus $\sigma _{13}=\sigma$; unless otherwise stated, all lengths are given in units of the monomer size $\sigma _{11}\equiv \sigma$.

In this work, the interactions between all pairs except between a sticky patch-sphere and a monomer are chosen to be purely repulsive (i.e. hard-sphere like) with a short-range cutoff distance $r_{ij}^c = \sigma _{ij} \times 2^{1/6}$. Two patches belonging to different H-NS molecules interact with each other when their center-to-center distance is within $r_{22}^c=0.2 \sigma$: $\sigma _{22} = 0.2/2^{1/6} \sigma \simeq 0.178\sigma$. If the interaction between each patch (small sphere in cyan on the left in Fig. 1) and a monomer were purely repulsive with the cutoff distance $r_{12}^c=0.6 \sigma$, $\sigma _{12}$ would be chosen to be $\sigma _{12} = r_{12}^c/2^{1/6}= 0.6/2^{1/6} \sigma \simeq 0.535\sigma$. Based on this, we choose $r_{12}^c = \sigma _{12} \times 2^{1/6}+ 0.085\sigma =0.685\sigma$ so that the interaction is attractive in the range $0.6< r < r_{12}^c\approx 0.685$. The simulation parameters specifying the interaction *U*(*r*) in Eq. (1) are listed in Table 1.

**Table 1:** Parameter values chosen for the Lennard-Jones (LJ) potential between interacting pairs in Eq. (1): $\text {monomers} \, (1)$, $\text {sticky patch-spheres} \, (2)$, $\text {core spheres} \, (3)$, and $\text {crowders} \, (4)$ (for the case $N=200$).

$\text {Pair}$	$\epsilon _{ij} [\epsilon ]$	$\sigma _{ij} [\sigma ]$	$r_{ij}^c [\sigma ]$
1-1	1.0	1.0	$2^{1/6}$
1-2	29.0	0.535	0.685
1-3	1.0	1.0	$2^{1/6}$
1-4	1.0	1.5	$1.5\times 2^{1/6}$
2-2	1.0	0.178	0.2
2-3	N/A	N/A	N/A
2-4	N/A	N/A	N/A
3-3	1.0	1.0	$2^{1/6}$
3-4	1.0	1.5	$1.5\times 2^{1/6}$
4-4	1.0	2.0	$2.0 \times 2^{1/6}$

Lengths and energy scales are given in units of $\sigma _{11}=\sigma$ and $\epsilon _{11}=\epsilon$. Except between (1) and (2), we use $r_{ij}^c = \sigma _{ij} \times 2^{1/6}$ (purely repulsive). Two patches belonging to different H-NS molecules interact with each other when their center-to-center distance is within $r_{22}^c=0.2\sigma$: $\sigma _{22} = 0.2 \sigma /2^{1/6} \simeq 0.178\sigma$. If the interaction between each patch and a monomer were purely repulsive with the cutoff distance $r_{12}^c=0.6 \sigma$, $\sigma _{12}$ would be chosen to be $\sigma _{12} = r_{12}^c/2^{1/6}= 0.6 \sigma /2^{1/6} \simeq 0.535\sigma$. With the choice $r_{12}^c = \sigma _{12} \times 2^{1/6}+ 0.085\sigma =\ 0.685\sigma$, however, the interaction between a patch sphere and a monomer becomes attractive in the range $0.6< r < r_{12}^c\approx 0.685$. All the parameter values used for the case $N=200$ are listed in the Table. For $N=2000$, we set $\sigma _{14}=2.5\sigma$, $\sigma _{34}=2.5\sigma$, and $\sigma _{44}=4.0\sigma$ (corresponding cutoff distances are $r_{14}^c = 2.5\sigma \times 2^{1/6}$, $r_{34}^c = 2.5\sigma \times 2^{1/6}$, and $r_{44}^c = 4.0\sigma \times 2^{1/6}$, respectively), but other parameter values remain the same as those chosen for $N=200$.

For $N=2000$, we set $\sigma _{14}=2.5\sigma$, $\sigma _{34}=2.5\sigma$, and $\sigma _{44}=4.0\sigma$ (the corresponding cutoff distances are $r_{14}^c = 2.5\sigma \times 2^{1/6}$, $r_{34}^c = 2.5\sigma \times 2^{1/6}$, and $r_{44}^c = 4.0\sigma \times 2^{1/6}$, respectively), but other parameter values remain the same as those chosen for $N=200$.

**Figure 3:**
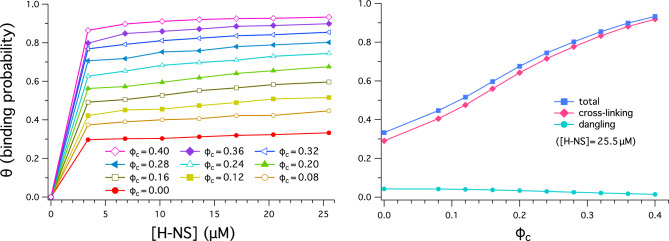
Binding probability $\theta$ of H-NS to a model chromosome as a function of $[\text {H-NS}]$ (on the left) and $\phi _c$ (on the right). (Left) As $[\text {H-NS}]$ increases, $\theta$ increases; it is expected to get saturated as $[\text {H-NS}]\rightarrow \infty$. Also note that $\theta$ is larger for larger $\phi _c$ for fixed $[\text {H-NS}]$. This observation suggests that the collapse of a chromosome-like polymer induced by crowding promotes H-NS binding possibly by enhancing their propensity for cross-linking between two sites on the polymer, as suggested in the graph on the right. (Right) This graph shows the fraction of bound H-NS in two binding modes: dangling (single-site binding) and cross-linking (two-site binding). The curves shown were obtained with a representative value of $[\text {H-NS}]=25.5 \, \mu \text {M}$. As $\phi _c$ increases, the fraction of cross-linking H-NS increases rather rapidly but the fraction of dangling H-NS decreases slowly. Also shown is the sum of the two, which increases with $\phi _c$. The dependence of H-NS binding displayed in this graph shows how the presence of crowders enhances the binding of cross-linking H-NS.

We set the strength of interaction between the H-NS patch and a monomer to $\epsilon _{12} = 29.0 \epsilon$. With this choice, the interaction energy *U*(*r*) in Eq. (1) evaluated at $r=r_\text{min} \approx 0.6$ becomes $U(r_\text{min}) \approx -8.65$ in units of $\epsilon =k_{{\text{B}}}T$, where $r_\text{min}$ is the distance at which the potential energy reaches its minimum. At this value, about a third of H-NS binds to DNA in the absence of crowders as evidenced later (see red line on the left in Fig. 3). The potential energy ($-8.65 \, k_{{\text{B}}}T$) resulting from our choice of $\epsilon _{12}$ is close to the binding free energy of H-NS to DNA at $20^\circ \text{C}$: $-21.09 \,{\mathrm{kJ/mol}} \simeq -8.51 \, k_{{\text{B}}}T$^33^. The strength of interaction between all other pairs is set to $\epsilon _{ij} = \epsilon$.

Each DNA monomer coarse-grains and represents about 7.35 hydrated B-DNA basepairs (bp). A natural choice for the monomer size is thus $\sigma =\sigma _{11}=2.5 \, \text {nm}$^2,24^. The diameter of both DNA and H-NS is $2.5 \, \text {nm}$; the size of a crowding particle is then $\sigma _c = 2\sigma =5.0 \, \text {nm}$^2^. All the particles are confined within a cubic box of side $50\sigma = 125 \, \text {nm}$ for $N=200$ and $110\sigma \simeq 270 \, \text {nm}$ for $N=2000$, respectively. As a result, the concentration of monomers in the cube is approximately $170\, \mu \text {M}$. The periodic boundary conditions are imposed on each side.

The volume fraction of crowding particles is in the range: $0\le \phi _c \lesssim 0.4$. The volume fraction of H-NS varies from 0 to $25.5 \, \upmu \text{M}$.

## Results

In our studies, both the volume fraction of crowders, $\phi _c$, and the number of H-NS, $N_\text{HNS}$, are key parameters. Following the simulation procedure described in Sect. "Computational modeling", we have first calculated chain size as a function of $\phi _c$ for various choices of $N_\text{HNS}$ and plotted the results in Fig. 2. Primarily, we have chosen $N=200$ and $N_\text {HNS}=0, 4, 8, 12, 16, 20, 30$, which correspond to H-NS concentrations $[\text {H-NS}] =0, 3.4, 6.8, 10.2, 13.6, 17.0, 25.5 \, \mu \text {M}$, respectively. The entire system is confined inside a cube of volume $125\!\times \! 125\! \times \! 125\,\text {nm}^3$, as discussed in Sect. "Computational modeling". In this work, $[\cdots ]$ denotes a molar concentration.

Displayed in Fig. 2 is the reduced chain size as a function of $\phi _c$: $R_g/R_{g0}$, where $R_g$ is the radius of gyration and $R_{g0}=R_g(\phi _c=0,[\text {H-NS}]=0)$. In all cases shown, the reduced polymer size decreases as $\phi$ increases; it decreases more rapidly for larger $[\text {H-NS}]$. For given $\phi _c$, it is smaller for larger $[\text {H-NS}]$. When $[\text {H-NS}]=25.5 \, \mu \text {M}$, for example, DNA is compacted by about $13 \%$ ($R_g/R_{g0} \approx 0.87$) and $46\%$ ($R_g/R_{g0} \approx 0.54$) for $\phi _c=0$ and 0.32, respectively.

Also shown in Fig. 2 are curves that represent a much longer chain: $N=2000$ confined in a cube ($270\!\times \! 270\! \times \! 270\,\text {nm}^3$). For this, we have chosen $[\text {H-NS}]=0, 25.5 \, \mu \text {M}$. When $[\text {H-NS}]=25.5 \, \upmu \text{M}$ and $\phi _c=0.32$, the polymer is condensed more effectively in reference to the corresponding short chain case: $R_g/R_{g0} \approx 0.34$. This trend persists even when $[\text {H-NS}]=0$; compare the solid curve with filled circles with the dashed curve with open circles. This points to the significance of chain length in chain compaction by biomolecular crowding. Nevertheless, the general trend observed with a shorter chain remains applicable to a longer chain, which is computationally more demanding. For the remainder of this work, we will only consider the short-chain case $N=200$.

As noted above, the chain size decreases more rapidly with $\phi _c$, when $[\text {H-NS}]$ is larger. This points to the synergy between crowders and H-NS. It appears to be consistent with the recent observation that H-NS enhances bacterial chromosome compaction by crowding effects^20,21^. As it turns out, H-NS not only enhances the depletion force between chain segments by enlarging the chain thickness, as assumed in Ref. ^20^, it also binds more tightly to a chromosome-like polymer for larger $\phi _c$, as if the presence of crowders increases the binding affinity of H-NS, as shown below. This interdependence is also implicated in crowder and H-NS synergetics.

We have examined further how the effects of H-NS and crowders are interrelated. Figure 3 shows the fraction of bound H-NS molecules to a ring polymer, denoted as $\theta$; if all H-NS molecules are bound in a “hypothetical” situation, $\theta =1$.

As shown in the graph on the left in Fig. 3, $\theta$ increases with increasing $[\text {H-NS}]$; it is expected to get saturated as $[\text {H-NS}]\rightarrow \infty$. In this graph, different colors represent various choices of $\phi _c$. For given $[\text {H-NS}]$, $\theta$ is larger for larger $\phi _c$, as if the presence of crowers enhances the binding affinity of H-NS for the polymer. This observation suggests that chain collapse induced by crowding promotes H-NS binding possibly by enhancing their propensity for cross-linking, as evidenced below.

It proves useful to decompose H-NS binding into the two binding modes: single-site or dangling and two-site binding; in the latter case, both the two binding sites of a H-NS dimer are occupied. In earlier studies^22,23^, two-site binding was further classified into *cis* and *trans*. If a H-NS dimer binds two distant genomic sites (e.g. more than two monomers or beads apart), it is in *trans*; otherwise, it is in *cis*. Cis binding requires bending of a H-NS dimer into a ‘U’ shape, which is suppressed in our modeling as in Ref.^24^. One can argue on physics grounds that this complication will not limit the physical picture presented in Fig. 2: cross-linking or bridging two adjacent monomers will not contribute toward chain compaction much more effectively than dangling. See below for additional discussions. In this work, two-site binding and cross-linking can be used interchangeably.

In the graph on the right in Fig. 3, the two binding modes, i.e. dangling and cross-linking are represented by different curves: solid lines with diamonds (cross-linking) and circles (dangling). The curves were obtained with a representative value of $[\text {H-NS}]=25.5 \, \mu \text {M}$. As $\phi _c$ increases, the fraction of cross-linking H-NS increases rather rapidly but the fraction of dangling H-NS decreases slowly. But the sum of the two, represented by the top curve, increases with $\phi _c$. The dependence of H-NS binding displayed in this graph shows how the presence of crowders enhances the binding of H-NS in cross-linking mode: crowding brings close otherwise distant monomers, making easier their cross-linking by H-NS.

We note from Fig. 3 that the presence of crowders enhances the binding of H-NS to a bacterial chromosome. Can H-NS binding in turn enhance chain compaction? Earlier studies suggest that this is indeed the case^20,21^: H-NS enhances the ability of crowders to collapse bacterial chromosomes. This was interpreted in terms of bound H-NS enlarging the polymer thickness^20^. While this mean-field approach serves its purpose, a more complete understanding would necessitate a systematic analysis of the effects of H-NS and crowders on chain compaction under controlled conditions.

**Figure 4:**
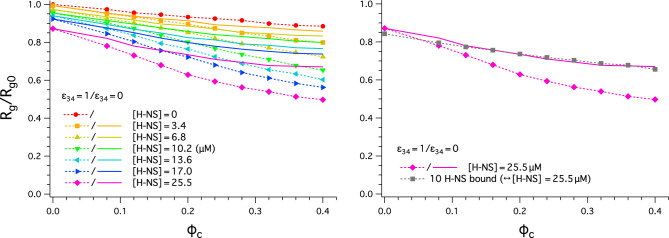
Synergy between H-NS and crowders in condensing a chromosome-like polymer. (Left) The curves in various colors represent different choices of $[\text {H-NS}]$. Two sets of curves are compared: the curves obtained with $\epsilon _{34} =1$ (solid lines with symbols) and for $\epsilon _{34}=0$ (dashed lines). Here, $\epsilon _{34}$ is the strength of the LJ potential between H-NS and crowders; $R_g$ is the radius of gyration and $R_{g0}$ is its unperturbed value in the absence of both crowders and H-NS. When $\epsilon _{34}=0$, H-NS is permeable to crowders. As a result, H-NS does not feel any steric hindrance caused by the surrounding crowders; two H-NS molecules will not experience any depletion force. The difference between the solid and dashed curves arises solely from the depletion force between H-NS molecules (included in the solid curves) and can be viewed as a quantitative measure of the synergy between H-NS and crowders. This difference is more pronounced for large $[\text {H-NS}]$ and increases with increasing $\phi _c$. (Right) The grey dashed curve with filled squares displayed was obtained with $\epsilon _{34} =1$ and 10 H-NS dimers anchored to randomly-chosen sites on the polymer. The same number of H-NS was bound, when $\phi _c=0$ and $[\text{HNS}]=25.5 \, \upmu \text{M}$ (Fig. 3). The curve was averaged over 10 independent choices of randomly-chosen binding sites. The consequence of fixing the number of bound H-NS is as significant as the depletion force between monomers enhanced by bound H-NS.

In the graph on the left in Fig. 4, two sets of curves are compared: the curves obtained with $\epsilon _{34} =1$ (dashed lines with symbols) and $\epsilon _{34}=0$ (solid lines); the curves in various colors represent different choices of $[\text {H-NS}]$. Here, $\epsilon _{34}$ is the strength of the LJ potential between H-NS and crowders; recall that $R_g$ is the radius of gyration and $R_{g0}$ is its unperturbed value in the absence of both crowders and H-NS. When $\epsilon _{34}=0$, H-NS is permeable to crowders: it can penetrate them. As a result, H-NS does not feel any steric hindrance caused by the surrounding crowders; two H-NS molecules will not experience any depletion force. The difference between the solid and dashed curves arises solely from the depletion force between H-NS molecules (solid curves) and can be viewed as a quantitative measure of the synergy between H-NS and crowders. This difference is more pronounced for large $[\text {H-NS}]$ and increases with increasing $\phi _c$.

The results in Fig. 3 capture the enhancement of H-NS binding by crowding. To examine its consequence on H-NS and crowder synergetics, in the graph on the right in Fig. 4, we have included a grey dashed curve with filled squares. For this, we chose $\epsilon _{34} =1$ as for the dashed line with diamonds from the left graph and anchored 10 H-NS molecules to randomly-chosen sites on the polymer; the curve was averaged over 10 independent choices of binding sites. The number of anchored H-NS chosen represents the case $[\text {H-NS}]=25.5 \, \upmu \text{M}$ in the absence of crowders ($\phi _c=0$). As shown in Fig. 3, when $\phi _c=0$, $\theta$ approaches 0.3, as $[\text {H-NS}]$ increases; $\theta \approx 0.33$ if $[\text{HNS}]=25.5 \, \upmu \text{M}$. We thus set the number of anchored H-NS molecules to the total number of bound H-NS: $0.33 \times 30 \approx 10$ (recall 30 H-NS molecules in a cube of volume $125\!\times \! 125\! \times \! 125\,\text {nm}^3$ corresponds to $[\text{HNS}]=25.5 \, \upmu \text{M}$). By fixing the number of bound H-NS at this value, the enhancement of H-NS binding induced by crowding effects as seen in Fig. 4 is turned off. The resulting curve in grey obtained with the parameters used is close to the solid curve in magenta obtained with $\epsilon _{34}=0$. Ignoring crowding-enhanced H-NS binding has as significant effects on chain compaction as ignoring H-NS-enhanced depletion forces between monomers.

As discussed earlier, our model does not distinguish between the two binding modes: *cis* and *trans*. H-NS dimers in *cis* mode would not contribute to chain compaction as efficiently as those in *trans* mode. In the absence of crowders, their effect on chain compaction is expected to be minimal, similarly to the effect of dangling H-NS. In the presence of crowders, H-NS dimers enhance chain compaction by crowders irrespectively of their binding mode. The degree of enhancement is expected to be insensitive to their binding mode, since the depletion free energy gain depends solely on the degree of overlapping between depletion layers for a given value of $\phi _c$, as shown on the right in Fig. 1. As a result, the essence of our finding in Fig. 4 will not reflect sensitively the coarse-grained nature of H-NS binding in this work.

A general picture emerging from Figs. 3 and 4 is that the synergetics between H-NS and crowders in condensing bacterial chromosomes is two-way one: H-NS binding enhances the depletion forces between chain segments^20,21^; the presence of crowders enhances the binding of H-NS to the chromosome, which in turn makes even stronger the depletion forces. Even when H-NS and crowders separately do not have a significant impact on chain compaction, the simultaneous presence of the two condenses a chromosome-like polymer appreciably better than what we would expect from the sum of the two individual effects. To be specific, consider the case $[\text {H-NS}]=25.5 \, \upmu \text{M}$ and $\phi _c=0.32$, and relate it to the corresponding H-NS-only or crowder-only case. The following inequality holds: $46\% \, \text {compaction (both)} > 23\% \, \text {compaction}$, which is a sum of $13\% \, \text {(H-NS only)}$ and $10\% \, \text {(crowder only)}$. This exemplifies the degree of H-NS and crowder synergetics in a quantitative manner.

**Figure 5:**
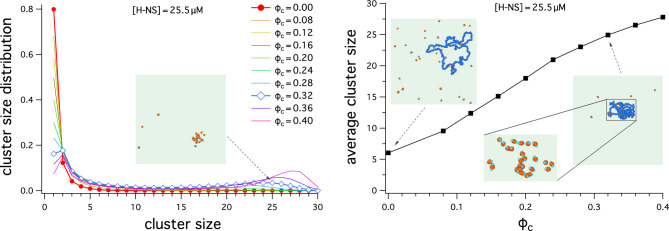
Cluster-size distribution (on the left) and average cluster size versus $\phi _c$, the volume fraction of crowders (on the right). The cluster size is measured by counting the number of H-NS core particles whose center-to-center distance is within $3\sigma$. (Left) When $\phi _c$ is in the range $0\le \phi _c \le 0.20$, the cluster-size distribution has a single peak around 2 or reaches its maximum at 1, while for $\phi _c> 0.20$, it has double peaks with the second peak located at a much larger value of the cluster size ($\approx 24$ for $\phi _c=0.32$), signalling the formation of a large cluster of H-NS particles. The snapshot shows such a cluster for $\phi _c= 0.32$; for simplicity, only H-NS is included. (Right) The average cluster size, obtained with $[\text {H-NS}]=25.5 \, \upmu \text{M}$, increases with increasing $\phi _c$, as expected from the graph on the left. The left and right snapshots included in this graph correspond to $\phi _c=0$ and $\phi _c=0.32$, respectively. The enlarged view captures several H-NS clusters, including the big one in the lower middle. For visual clarity, in all the snapshots in both graphs, the crowders are hidden and the patches are sticking out of the core.

**Figure 6:**
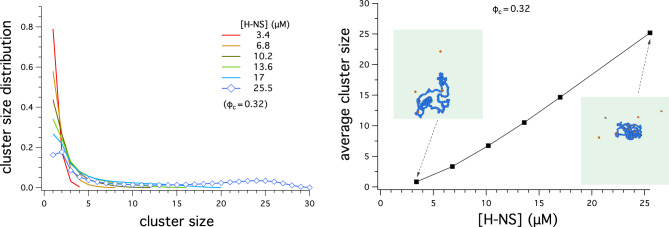
Cluster-size distribution (on the left) and average cluster size versus $[\text {H-NS}]$ (on the right). For this, we have chosen $\phi _c=0.32$. When the center-to-center distance of H-NS core particles is within $3\sigma$, they are considered as forming a cluster. (Left) Except for $[\text {H-NS}]=25.5 \, \upmu \text{M}$, the cluster-size distribution is single-peaked at 1; when $[\text {H-NS}]=25.5 \, \upmu \text{M}$, it has double peaks with the second peak located at a much larger value of the cluster size ($\approx 24$), signalling the formation of a large cluster of H-NS particles. (Right) The average cluster size, obtained with $\phi _c=0.32$, increases with increasing $[\text {H-NS}]$, as expected from the graph on the left. The snapshots included in this graph correspond to $[\text {H-NS}]=25.5 \, \upmu \text{M}$ and $\phi _c=0.32$. For visual clarity, in all the snapshots in this figure, the crowders are hidden and the patches are sticking out of the core.

We have also considered the clustering of H-NS driven by depletion forces. Figures 5 and 6 summarize the results for H-NS clustering: the cluster-size distribution (on the left) and the average cluster size (on the right). When the center-to-center distance between the two neighboring H-NS cores is within $3\sigma$, they are viewed as forming a cluster. Here, we do not differentiate between different clustering configurations, e.g. a linear array of three H-NS particles and three H-NS forming a triangle; both clusters have the same size, i.e. 3.

The graph on the left in Fig. 5 shows the cluster-size distribution as a function of the cluster size. When $\phi _c$ is in the range $0\le \phi _c \le 0.20$, the cluster-size distribution has a single peak around 2 or reaches its maximum at 1. In contrast, for $\phi _c> 0.2$, it has double peaks with the second peak located at a much larger value of the cluster size, signalling the formation of a large cluster of H-NS particles. The snapshot shows such a cluster for $\phi _c= 0.32$; for simplicity, only H-NS is included.

The graph on the right in Fig. 5 displays the average cluster size, obtained with $[\text {H-NS}]=25.5 \, \upmu \text{M}$. It increases with increasing $\phi _c$, as expected from the graph on the left. The left and right snapshots included in this graph correspond to $\phi _c=0$ and $\phi _c=0.32$, respectively. The enlarged view captures several H-NS clusters, including the big one in the lower middle. For visual clarity, in all the snapshots in both graphs, the crowders are hidden and the patches are sticking out of the core.

Shown in Fig. 6 is the cluster-size distribution for various values of $[\text {H-NS}]$ (on the left) and the average cluster size as a function of $[\text {H-NS}]$ (on the right); the volume fraction of crowders is held fixed at $\phi _c=0.32$.

As shown on the left in Fig. 6, the cluster-size distribution is single-peaked at 1 except for $[\text {H-NS}] =25.5 \, \upmu \text{M}$; when $[\text {H-NS}] =25.5 \, \upmu \text{M}$, however, it develops a second peak at a large value of the cluster size ($\approx 24$).

The graph on the right in Fig. 6 suggests that the average cluster size, obtained with $\phi _c=0.32$, increases with increasing $[\text {H-NS}]$, as expected from the graph on the left. The snapshots included in this graph correspond to $[\text {H-NS}] =3.4 \, \upmu \text{M}$ (left inset) and $25.5 \, \upmu \text{M}$ (right inset).

H-NS clustering suggested in Figs. 5 and 6 is analogous to oligomerization of H-NS^4,6^. However, the former is not conclusive for the latter, since our simplified model leaves out potentially relevant biological details such as possible biomolecular interactions between H-NS proteins and chain stiffness. Nevertheless, they point to the possibility that crowding effects can be involved in the oligomerization of H-NS proteins; they also complement the finding that bridging-induced attraction drives H-NS clustering^24^.

## Discussions

Polymer physics has proven to be useful for advancing our understanding of chromosome organization and its impact on their biological functions^2,9–11,13,24–28^. Our work expands its repertory by including the effects of the cross-linking protein H-NS in addition to those of biomolecular crowders. The computational model employed in this work clarifies the cooperative nature of their effects on chromosome compaction.

In our simulations, H-NS is modeled as a triplex consisting of a core sphere and two small patch spheres, which are diagonally positioned inside the core. The patch spheres can bind to a (chromosome-like) ring polymer with characteristic binding energy. In this work, H-NS binding is further classified into two binding modes: dangling and cross-linking (or trans). Because of the way it is modeled, H-NS does not show a *cis* configuration, in which the two binding sites of a H-NS dimer bind simultaneously to DNA sites nearby (e.g. within a few monomers)^22,23^. *Cis* binding requires bending of a H-NS dimer into a ‘U’ shape and is not realized in our modeling. In the absence of crowders, however, H-NS dimers in *cis* binding does not contribute to DNA compaction. In a crowded medium, they can enhance DNA compaction by crowding effects, similarly to what we expect from dangling H-NS dimers. As a result, the addition of *cis* binding in our consideration will not change the general picture of DNA compaction by H-NS and crowders.

Along the line of what is discussed above, it is worth mentioning that H-NS binding and its impact on DNA, carrying a negative charge, depend on the presence of $\text{Mg}^{2+}$^4,34,35^: at a low concentration of $\text{Mg}^{2+}$ ($< 2 \, \text{mM}$), H-NS coats DNA without looping it; at a high concentration ($> 5 \, \text{mM}$), it can bridge the DNA and thus contribute to DNA compaction. $\text{Mg}^{2+}$ can reduce the repulsion between the backbone charges on DNA or even turn the repulsion into attraction^36^. As a result, the presence of $\text{Mg}^{2+}$ enhances the propensity of H-NS to cross-link DNA and thus that of clustering. This effect can be taken into account at least implicitly by adjusting the simulation parameters such as $\epsilon _{ij}$ and $r^c_{ij}$.

The model used in this work clearly captures the synergy between H-NS and crowders in condensing the polymer beyond the recent effort, in which the effect of H-NS binding is viewed as thickening the chromosome^20^. The results presented in this work suggest a two-way synergetics between H-NS and crowders: if the presence of crowders enhances H-NS binding to a chromosome-like polymer by increasing their propensity for chain cross-linking, the presence of H-NS makes crowding effects more efficient by locally enlarging chain segments, analogous to what was observed with a heterogeneous polymer in a crowded medium^9^.

Besides H-NS and other NAPs (e.g. HU and IHF), RNA polymerases are known to be key players in organizing bacterial chromosomes^2,37,38^. It has been shown that the binding of RNA polymerases to *E. coli* chromosomes especially under fast grow conditions induces clustering of transcription-active units^2^. Recent computer simulations support this picture^9^. Both H-NS and RNS polymerases enhance chromosome compaction by crowding effects. In a more general perspective, depletion forces induced by biomolecular crowding are size-dependent^2,12,13,18^. They are stronger between bigger objects (e.g. H-NS bound sites on a chromosome).

It is worth appreciating the clear difference between H-NS and RNA polymerases (or some other chromosome-associated proteins that do not induce cross-linking). H-NS not only enhances depletion forces between chromosome segments but also cross-links two sites on a chromosome.

The results reported here tend to illustrate the dual or multiple roles of biomolecules. Beyond their biological specificity, they are physical entities, exerting excluded volume interactions or causing steric hindrance to other molecules, as is the case for H-NS. Similar analysis of other chromosome-associated proteins is desired for a fuller picture.

In this work, we have focused our effort on examining the equilibrium behavior of chromosome compaction by H-NS and crowders. Accordingly, the results for H-NS binding in Fig. 3 left out the dynamical aspects of H-NS binding/unbinding and cross-linking. In light of studies on ‘facilitated’ (concentration-accelerated) dissociation of chromosome-associated proteins (e.g. Fis)^39,40^, it will be useful to extend our equilibrium effort toward modeling association/dissociation and cross-linking dynamics of H-NS in a crowded medium.

Finally, we would like to add that biomolecular crowding can influence other biological processes such as protein folding/aggregation, gene regulation, and cell growth^2,41–43^. We hope that our work will inspire more investigation into the intriguing roles of crowders in biological processes.

## Data Availability

The datasets used and analysed during the current study are available from the corresponding authors on reasonable request.

## Code availability

The codes used to generate the datasets in this work are available at the following link: https://github.com/yjung-github/H-NS-crowder.
